# Sub-1-min relaxation-enhanced non-contrast non-triggered cervical MRA using compressed SENSE with deep learning reconstruction in healthy volunteers

**DOI:** 10.1186/s41747-025-00560-7

**Published:** 2025-02-18

**Authors:** Jan Paul Janssen, Kenan Kaya, Robert Terzis, Robert Hahnfeldt, Roman Johannes Gertz, Lukas Goertz, Stephan Skornitzke, Juliana Tristram, Thomas Dratsch, Cansin Goezdas, Christoph Kabbasch, Kilian Weiss, Lenhard Pennig, Carsten Herbert Gietzen

**Affiliations:** 1 Institute for Diagnostic and Interventional Radiology, Faculty of Medicine and University Hospital Cologne, University of Cologne, Cologne, Germany; 2 Philips GmbH, Hamburg, Germany

**Keywords:** Carotid arteries, Deep learning, Healthy volunteers, Magnetic resonance angiography, Neck

## Abstract

**Background:**

We evaluated the acceleration of a three-dimensional isotropic flow-independent magnetic resonance angiography (MRA) (relaxation-enhanced angiography without contrast and triggering, REACT) of neck arteries using compressed SENSE (CS) combined with deep learning (adaptive intelligence, AI)-based reconstruction (CS-AI).

**Methods:**

Thirty-four volunteers received 3-T REACT MRA, acquired threefold: (i) CS acceleration factor 7 (CS7), scan time 1:20 min:s; (ii) CS acceleration factor 10 (CS10), scan time 0:55 min:s; and (iii) CS-AI acceleration factor 10 (CS10-AI), scan time 0:55 min:s. Two radiologists rated the image quality of seven arterial segments and overall image noise. Additionally, a pairwise forced-choice comparison was conducted. Apparent signal-to-noise ratio (aSNR) and contrast-to-noise ratio (aCNR) were measured, and image sharpness was assessed using the edge-rise distance (ERD). Multiple *t*-tests and nonparametric tests with Bonferroni correction were performed for comparison to CS7 as the reference standard.

**Results:**

Compared to CS7, CS10 showed lower image quality (*p* < 0.001) while CS10-AI obtained higher scores (*p* = 0.010). Image noise was similar between CS7 and CS10 (*p* = 0.138) while CS10-AI yielded a lower noise (*p* = 0.008). Forced choice revealed preferences for CS7 over CS10 (*p* < 0.001), but no preference between CS7 and CS10-AI (*p* > 0.999). Compared to CS7, aSNR and aCNR were lower in CS10 (*p* < 0.001) and the ERD was longer (*p* = 0.004), while CS10-AI provided better aSNR and aCNR (*p* = 0.001) and showed no difference in ERD (*p* = 0.776).

**Conclusion:**

Sub-1-min CS-AI cervical REACT MRA was acquired without compromising image quality.

**Relevance statement:**

The implementation of a fast and reliable non-contrast MRA has the potential to reduce costs and time while increasing patient comfort and safety. Clinical studies evaluating the diagnostic performance for stenosis or dissection are needed.

**Trial registration:**

DRKS00030210 (German Clinical Trials Register; https://drks.de/)

**Key Points:**

- Deep learning reconstruction enables sub-1-min non-contrast-enhanced MRA of extracranial arteries.
- Acceleration without deep learning reconstruction causes inferior image quality.
- Acceleration with deep learning reconstruction exceeds, in part, the clinical standard.

**Graphical abstract:**

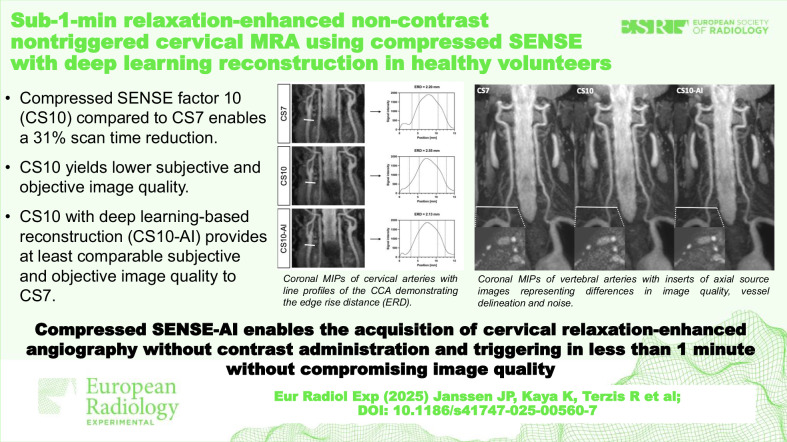

## Background

In acute stroke imaging, CT is preferred in time-critical cases due to its speed and availability. Magnetic resonance imaging (MRI), however, provides enhanced diagnostic capabilities in less urgent scenarios and is the focus of this study [1, 2]. Combination with contrast-enhanced magnetic resonance angiography (CE-MRA) of the neck provides a radiation-free and noninvasive depiction of the cervical arteries for the detection of potential stroke etiologies *e.g.*, relevant internal carotid artery (ICA) stenosis, with 66–97% sensitivity 91–96% specificity, thrombus, or dissection. This is relevant for guiding therapeutic decisions, such as thrombectomy, by ruling out major stenoses or occlusions [3–6].

Nevertheless, there are constraints associated with gadolinium-based contrast agents, *e.g.*, allergic reactions, risk of nephrogenic systemic fibrosis in end-stage renal disease, potential mistiming resulting in non-diagnostic studies, and unknown long-term effects of gadolinium deposition in the body [7, 8]. In addition, patient preparation for CE-MRA can be time-consuming and hamper clinical workflows. Consequently, there is a need for non-contrast MRA that can reliably visualize vascular pathology. However, these techniques require long acquisition times of up to seven minutes, hampering their use in clinical routine and emergency imaging [9–13].

Recently, a novel non-contrast MRA technique, “relaxation-enhanced angiography without contrast and triggering” (REACT), has been introduced [14]. REACT employs inversion-recovery and T2 preparation prepulse with Dixon readout to acquire a flow-independent three-dimensional (3D) isotropic non-contrast MRA [14]. It proved useful for imaging the cervical arteries in acute ischemic stroke, yielding to CE-MRA comparable image quality with high sensitivity and specificity for ICA stenoses and potentially improved depiction of adjacent plaques [15–17]. Despite using compressed sensitivity encoding (SENSE) (CS), a method combining parallel imaging and compressed SENSE, for the acceleration of image acquisition, REACT still requires a scan time between 1:20 min:s (with acceleration factor 7) [17] and 2:46 min:s (with acceleration factor 4) [15, 16], being longer than currently used CE-MRA [8, 9]. As indicated in these prior studies [15–17] further acceleration reduces image quality and might hamper diagnostic performance due to increased image noise and undersampling artifacts [17, 18].

Deep learning (DL)-based reconstruction may offer a solution to overcome these limitations [19]. Recently, Pezotti et al introduced compressed SENSE with adaptive intelligence reconstruction−adaptive-CS-Net (CS-AI) as part of the “fastMRI challenge 2019”, enabling a substantial acceleration without compromising image quality [19].

In this way, a substantial reduction in the acquisition time for cardiac cine balanced steady-state free precession (bSSFP) or non-contrast MRA of the coronary arteries has already been demonstrated [20, 21]. However, the potential for further acceleration of REACT has not yet been investigated.

The objective of this study was to evaluate the further acceleration of REACT of the cervical arteries using CS-AI to enable the acquisition of REACT in a time that is equal to CE-MRA without compromising image quality. Therefore, the subjective and objective image quality of REACT acquired with acceleration factor 10 with DL-based reconstruction (CS10-AI) and of REACT with acceleration factor 10 acquired without DL-based reconstruction (CS10) was compared to the currently used clinical standard, *i.e*, REACT with acceleration factor 7 acquired without DL-based reconstruction (CS7) in a cohort of healthy volunteers at 3 T.

## Methods

### Study design and population

This prospective single-center study was conducted in accordance with the ethical standards of the 1964 Declaration of Helsinki and its subsequent amendments and was approved by our institutional review board on March 23, 2022 (reference number: 20-1296_1). The study was registered in the German Clinical Trials Register on September 15, 2022 (DRKS00030210).

Recruitment of 38 volunteers and data acquisition took place between July 2023 and April 2024. Written informed consent was obtained from all participants. The inclusion criterion was an age of over 18 years. Participants who exhibited general contraindications to MRI (*e.g*., pacemaker, claustrophobia), inability to provide informed consent, pregnancy, obesity (body weight > 150 kg), or refusal of the consent to report incidental findings were excluded. Prior to image acquisition, the age, height, weight, gender, and cardiovascular risk factors, were recorded.

### MRI acquisition

A commercially available 3-T MRI system (Philips MR 7700, Philips Healthcare, Best, The Netherlands) with a 20-channel coil for head and neck imaging was used. All volunteers were placed supine, headfirst on the table. The k-space acquisition scheme of REACT for both the CS and the CS-AI acquisitions was based on a variable density incoherent undersampling pattern with a more densely sampled k-space center. [22]. Three acquisitions were conducted using the same acquisition and post-processing pipeline: (i) REACT with CS7 (fixed scan time 1:20 min:s, representing the current clinical standard [17]); (ii) REACT with CS10 (fixed scan time 0:55 min:s); and (iii) REACT with CS10-AI (fixed scan time 0:55 min:s).

REACT is a nontriggered, 3D isotropic flow-independent sequence based on T2 preparation and inversion-recovery pulses (allowing the enhancement of the native blood signal) followed by a 3D 2-point Dixon readout with flexible echo times containing a 7-peak fat model (mDIXON XD, Philips Healthcare) to suppress the fat and background signal [14]. Data acquisition was performed in the coronal plane. Given the known fat–water swapping artifacts of the Dixon readout [15–17], water-only, in- and out-of-phase images were reconstructed [23]. Table 1 shows the scan parameters.

**Table 1:** Scan parameters of non-contrast MRA sequences

Sequence [reference(s)]	REACT [15, 16]	REACT	REACT	3D QISS [33]	CE-MRA [15–17]
Acceleration	CS4	CS7	CS10/ CS10-AI	4-fold (DNN-based)	CS6
Scan time (min:s)	02:46	01:20	00:55	01:40	1:08
k-space trajectory	Cartesian	Cartesian	Cartesian	Radial	Cartesian
Acquisition orientation	Coronal	Coronal	Coronal	Axial	Coronal
Acquired voxel size (mm^3^)	1.5 × 1.5 × 1.5	1.5 × 1.5 × 1.5	1.5 × 1.5 × 1.5	0.86 × 0.86 × 1.30	0.63 × 0.63 × 0.63
Reconstructed voxel size (mm^3^)	0.625 × 0.625 × 0.75	0.69 × 0.69 × 0.75	0.69 × 0.69 × 0.75	0.43 × 0.43 × 0.65	0.5 × 0.5 × 0.5
Field of view, FH × RL × AP (mm^3^)	320 × 400 × 80	300 × 300 × 80	300 × 300 × 80	288.6 × 300 × 300	320 × 280 × 80
T2 preparation (ms)/refocusing pulses	50/4	50/4	50/4	–	–
Repetition time (ms)	4.3	7.0	7.0	9.9	6.1
Echo times (ms)	1.45/2.6	1.93/4.5	1.93/4.5	1.6/3.7/5.7	1.96
Flip angle (°)	15	12	12	–	40

*3D* Three dimensional, *AP* Anterior–posterior, *CE-MRA* Contrast-enhanced magnetic resonance angiography, *CS7* Compressed SENSE (CS) acceleration factor 7, *CS10* CS acceleration factor 10, *CS10-AI* CS10 with adaptive-CS-net reconstruction, *DNN* Deep neural network, *FH* Feet-head, *QISS* Quiescent interval slice-selective, *REACT* Relaxation-enhanced angiography without contrast and triggering, *RL* Right-left

### MRI reconstruction

Standard CS and CS-AI reconstructions were performed using standard hardware as provided by the manufacturer of the MRI system with reconstruction times of 15 s and 20 s, respectively. Both reconstruction algorithms used in this study are CE-certified and USA Food and Drug Administration-approved, ensuring compliance with European and USA regulatory standards for clinical application.

The CS reconstruction employs a nonlinear iterative reconstruction combining SENSE-based parallel imaging with a regularized, iterative L1 norm minimization assuring data consistency and image sparsity in the wavelet domain [22, 24].

The CS-AI reconstruction (SmartSpeed, Philips Healthcare) enhances the conventional CS reconstruction by incorporating a convolutional neural network (“Adaptive-CS-Net”), an advancement of the DL-based “Iterative shrinkage-thresholding algorithm”−ISTA network which has been previously proposed [25]. The network integrates multiscale sparsification in a problem-specific learnable manner. This convolutional neural network-based sparsification approach is combined with the CS image reconstruction in an unrolled iterative reconstruction scheme and therefore, ensures data consistency and takes imaging priors such as coil sensitivity distribution, location of the image background, and other information into account. Training data from approximately 740,000 images with different anatomies, contrasts, and field strengths (1.5 T or 3 T) were used to adapt the algorithm [19].

### Subjective image quality

Image analysis was performed using a commercially available image viewer (DeepUnity Diagnost, release 1.1.1.1, Dedalus Healthcare Systems Group, Bonn, Germany). Source images and maximum intensity projections (water-only; slice thickness of 6 mm, gap of 0 mm) with optional window leveling and angulation were assessed. A radiologist in training with two years (R1) and a board-certified radiologist with six years (R2) of experience in MRA evaluated the images (CS7, CS10, and CS10-AI) independently in separate sessions and in random order. They were blinded to the imaging and reconstruction protocol, as well as volunteer data. Prior to evaluation, both readers were instructed by a board-certified neuroradiologist (18 years of experience in MRA) regarding the sufficient evaluation of image data using exemplary images of different grades of ICA stenosis and distinct levels of image quality. Randomization was performed using the “RAND” function in Microsoft Excel (Version 16.88, Microsoft, Redmond, WA, USA).

The following vessel sections were evaluated regarding image quality: aortic arch and its adjacent branches; bilateral common carotid artery (CCA); bilateral ICA, extracranial segment, C1; bilateral ICA, petrous segment, C2; bilateral vertebral artery, segments V1-3. All segments were rated using a 7-point Likert scale (1 = non-diagnostic; 2 = poor; 3 = sufficient; 4 = moderate; 5 = good; 6 = very good; 7 = excellent). Additionally, the overall image noise was rated on a 5-point Likert scale (1 = non-diagnostic; 2 = high noise; 3 = moderate noise; 4 = low noise; 5 = no impact on image quality).

For pairwise forced-choice comparison, two images of each subject were presented side by side to the blinded readers who had to choose which image they preferred to rule out vascular pathologies. The sequence combinations examined in random order were CS7 *versus* CS10, CS7 *versus* CS10-AI, and CS10 *versus* CS10-AI.

#### Anatomical variants

Furthermore, R1 and R2 were advised to assess MRA datasets for the presence of vascular findings such as anatomical variants or pathologies.

#### Fat–water swapping artifacts

Water maps of REACT were evaluated by R1 for the presence of fat–water swapping artifacts, as well as the corresponding signal of the in-phase image at the respective signal loss of the water map, if present [15–17].

### Objective image quality

A radiologist in training with three years of experience in MRA (R3) assessed the apparent signal-to-noise ratio (aSNR) and apparent contrast-to-noise ratio (aCNR) by placing regions of interest in REACT (water-only) source images. Signal intensity of the vessels was measured at 3 cm proximal (CCA) and 3 cm distal (ICA) to the carotid bifurcation on both sides. Additionally, a region of interest was placed at each measurement level in the adjacent sternocleidomastoid muscle to determine the background noise.

The following formulas were used [15]:

$${{{\rm{aSNR}}}}=\frac{{{{\rm{SI}}}}_{v}}{{\sigma }_{{{{\rm{SI}}}}_{m}}}{{{\rm{aCNR}}}}\,=\,\frac{({{{\rm{SI}}}}_{v}-{{{\rm{SI}}}}_{m})}{{\sigma }_{{{{\rm{SI}}}}_{m}}}$$

where ${{SI}}_{v}$ is the signal intensity of the measured vessel, ${{{\rm{SI}}}}_{m}$ is the signal intensity of the adjacent muscle and ${\sigma }_{{{{\rm{SI}}}}_{m}}$ being the corresponding standard deviation of ${{{\rm{SI}}}}_{m}$. Mean values of aCNR and aSNR for both sides and each level were calculated and used for further analysis.

**Fig. 1:**
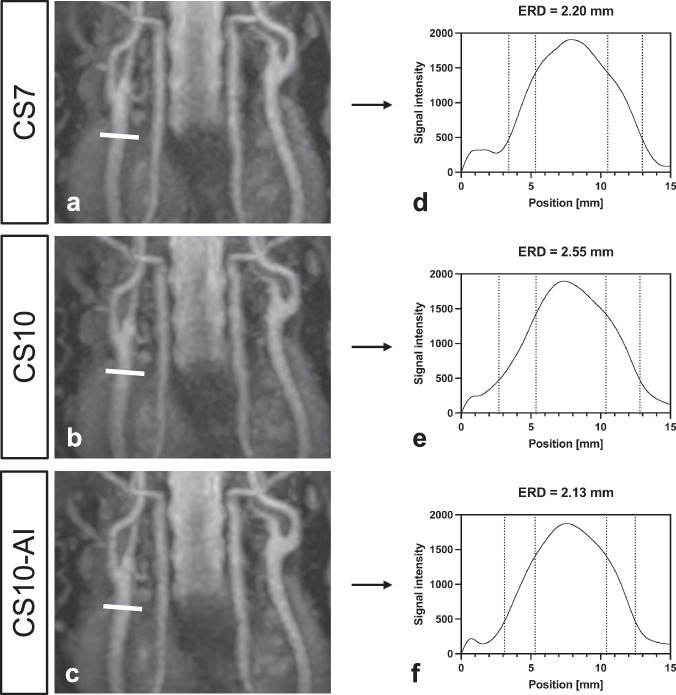
Determination of the edge-rise distance (ERD) on the right CCA as shown in a 51-year-old male volunteer (maximum intensity projections in coronal plane, slice thickness 30 mm, acquired using compressed SENSE acceleration factor 7 (CS7; **a**), CS10 (**b**), and CS10 with Adaptive-CS-Net reconstruction (CS10-AI; **c**). The ERDs are derived from the signal intensity profiles (**d**–**f**) placed perpendicular across the vessel lumen in source images (white line). To ascertain the mean ERD of the medial and lateral vessel wall, the difference between the full width at 25% of the maximum and the full width at 75% of the maximum is calculated. In the corresponding diagrams, the dotted vertical lines represent the medial and lateral points at 25% (outer lines) and 75% (inner lines) of the maximum signal intensity, which are used in calculating the ERD on the signal intensity profile. A shorter ERD indicates a sharper delineation of the transition between the vessel and surrounding tissue

Additionally, the edge rise distance (ERD) was determined as a measure of edge sharpness, which is critical for accurate vascular delineation in angiographic imaging (Fig. 1). Therefore, a 15-mm long line profile was drawn in an orthogonal orientation to the right CCA through the center of the vessel in the coronal source image. The intensity values were exported and interpolated with a cubic line function and ERD was calculated as follows [26]:

$${{{\rm{ERD}}}}=\frac{{{\rm{FW}}}25 \% M-{FW}75 \% M}{2}$$

where FW25%M and FW75%M are the full widths at 25% and 75% of the maximum intensity, respectively. A short ERD implies an effective separation between the vessel signal and the background noise, thereby indicating high image sharpness.

### Statistical analysis

Statistical analysis was performed with R v. 4.0.2 and the open-source software RStudio (posit.co, accessed on 12 January 2024). GraphPad Prism version 10.2.3 for Mac OSX (GraphPad Software) was used for data visualization. Categorical variables are presented as frequencies and corresponding percentages. Continuous variables are indicated as the mean ± standard deviation. Subjective ratings are presented as frequencies and corresponding percentages and median with interquartile range. Normal distributions were checked with the Shapiro–Wilk test. Differences were compared with Wilcoxon signed-rank tests (if not normally distributed, *e.g*., subjective ratings) or paired *t*-tests (if normally distributed, *e.g*., aSNR, aCNR, and ERD) and calculated per reader, as well as by combining the measurements of all readers. Kendall’s coefficient of concordance (*W*) was calculated to assess interobserver agreement for subjective image quality (0.01–0.2 slight, 0.21–0.4 fair, 0.41–0.6 moderate, 0.61–0.8 substantial, and 0.81–0.99 almost perfect) [27]. Pairwise forced choices were pooled for both readers, and differences between choices were compared with the binomial test, which is appropriate for binary data. Agreement between readers was assessed by Cohen κ (0.01–0.2 slight, 0.21–0.4 fair, 0.41–0.6 moderate, 0.61–0.8 substantial, and 0.81–0.99 almost perfect) [27]. For all tests, a two-tailed *p*-value < 0.05 was considered significant and *p*-values were adjusted by Bonferroni correction to address Type I error due to multiple comparisons.

*A priori* sample size was calculated using G*power 3.1.9.6. At least 34 subjects were required to detect a medium effect (Cohen *d* = 0.5) with an α error of 0.05 and a power of 0.80. A medium effect size was chosen as the benchmark because it represents a balance between statistical sensitivity and clinical relevance.

## Results

### Study population and baseline characteristics

Thirty-eight volunteers received REACT of the extracranial arteries. Four participants had to be excluded due to incomplete scan data. Consequently, 34 subjects were included (median age 59, interquartile range 54–62 years; 14 males; body mass index 25.03 ± 3.80 kg/m^2^, mean ± standard deviation). Information on the cardiovascular risk factors of the volunteers is provided in Table 2.

**Table 2:** Characteristics of the volunteers

Age, (years, median [IQR])	59 [54–62]	
Height, (cm, mean *±* SD)	172.0 ± 9.1	
Weight, (kg, mean *±* SD)	74.2 ± 12.8	
Body mass index, (kg/m^2^, mean *±* SD)	25.1 ± 3.8	
	Number	%
Gender		
Female	20	58.8
Male	14	41.2
Cardiovascular risk factors		
Hypertension	10	29.4
Diabetes mellitus	0	0.0
Smoking	4	11.8
Coronary artery disease	1	2.9

*IQR* Interquartile range, *SD* Standard deviation

### Subjective image quality

Each reader evaluated 68 datasets (34 each for CE-MRA and REACT), resulting in 476 evaluated arterial segments. Table 3 provides detailed results of the subjective image quality assessment pooled for both readers.

**Table 3:** Comparison of subjective image quality and noise scores pooled for both readers, as well as their interobserver agreement

	CS7	CS10	*p*-value	CS10-AI	*p*-value	*W* (*p*-value)
Image quality	Median [IQR]	Median [IQR]		Median [IQR]		
Overall	5.07 [4.52–5.43]	4.71 [4.38–5.11]	**<** **0.001**	5.29 [4.88–5.43]	**0.010**	0.88 (< **0.001**)
Aortic arch	4.25 [4–5]	4 [4–5]	0.120	5 [4.5–5.75]	**0.004**	0.77 (< **0.001**)
Carotid overall	6.25 [5.75–6.67]	5.92 [5.33–6.33]	< **0.001**	6.33 [6–6.67]	0.228	0.78 (< **0.001**)
CCA	6 [5–6]	5 [5–6]	**0.003**	6 [5.5–6]	0.160	0.71 (0.008)
ICA, C1	6.75 [6–7]	6 [5.5–7]	**<** **0.001**	7 [6–7]	0.316	0.70 (**0.006**)
ICA, C2	6 [6–7]	6 [5.5–7]	**0.002**	6.5 [6–7]	> 0.999	0.75 (**0.002**)
Vertebral overall	4.33 [3.33–4.67]	4 [3.38–4.33]	**<** **0.001**	4.25 [3.67–4.67]	0.189	0.83 (< **0.001**)
V1	4 [3–4.75]	3 [2.63–4]	**0.002**	3.75 [3–5]	> 0.999	0.86 (< **0.001**)
V2	4 [3.5–5]	4 [3.63–4.88]	0.064	4.5 [4–5]	0.518	0.82 (< **0.001**)
V3	4 [4.5–5]	4 [4–4.88]	0.053	4.5 [4–5]	0.507	0.73 (**0.003**)
Noise	4 [4–4]	4 [3.5–4]	0.138	4.25 [4–5]	**0.008**	0.59 (0.206)

Bold indicates statistical significance; *p*-values (Wilcoxon test, Kendall’s *W*) were adjusted by Bonferroni correction to address Type I error due to multiple comparisons

*CCA* Common carotid artery, *CS7* Compressed SENSE acceleration factor 7, *CS10* CS acceleration factor 10, *CS10-AI* CS10 with adaptive-CS-Net reconstruction, *ICA C1* Extracranial segment of the internal carotid artery, *ICA C2* Petrous segment of the internal carotid artery, *IQR* Interquartile range, *V1–3* Segments of the vertebral artery

For all arteries combined and compared to CS7 (5.07 [4.52–5.43]), CS10 showed lower image quality scores (4.71 [4.38–5.11]; *p* < 0.001) while CS10-AI achieved higher results (5.29 [4.88–5.43]; *p* = 0.010). In this context, CS10-AI achieved a score of ≥ 5 more frequently than CS7 at the aortic arch (67.7% [23 of 34 participants] *versus* 44.1% [15 of 34]; *p* = 0.004), while the other segments showed no significant difference. In contrast, CS10 achieved a score of ≥ 5 less frequently than CS7 in all segments, with segment V1 (11.8% [4 of 34] *versus* 26.5% [9 of 34]; *p* = 0.002) and all segments of the extracranial carotid arteries (combined: 87.3% [89 of 132] *versus* 96.1% [98 of 132]; *p* < 0.001) scoring significantly lower. Image noise was rated similar between CS7 and CS10 (*p* = 0.138) while CS10-AI yielded a lower noise than CS7 (*p* = 0.008). Overall, the concordance between both readers was substantial to almost perfect regarding image quality (*W* = 0.88; range: *W* = 0.71 in the C1 segment to *W* = 0.86 in the V1 segment), only noise ratings showed a moderate agreement with *W* = 0.59.

Pairwise forced choices revealed a preference for CS7 over CS10 (*p* < 0.001), a preference for CS10-AI over CS10 (*p* < 0.001), but no preference between CS7 and CS10-AI (*p* > 0.999). A comparison of the individual readers’ decisions between CS7 and CS10-AI revealed that the less experienced radiologist favored CS10-AI in 18 out of 34 cases, while the experienced radiologist made this decision in 19 out of 34 cases. However, both readers diverged in their decisions in 11 out of 34 cases, resulting in a slight interobserver agreement (*κ* = 0.35). Decisions combined for both readers are shown in Table 4.

**Table 4:** Forced decision results pooled for both readers with the corresponding interobserver agreement

	Both CS7 (*n*, %)	Both CS10 (*n*, %)	Both CS10-AI (*n*, %)	Draw (*n*, %)	*p*-value	*κ* (*p*-value)
CS7 *versus* CS10	30 (88.2)	2 (5.9)	–	2 (5.9)	**<** **0.001**	0.63 (< **0.001**)
CS7 *versus* CS10-AI	10 (29.4)	–	13 (38.2)	11 (32.4)	> 0.999	0.35 (0.128)
CS10 *versus* CS10-AI	–	0	31 (91.2)	3 (8.9)	**<** **0.001**	-0.05 (> 0.999)

Bold indicates statistical significance. *p*-values (binomial test, Cohens κ) were adjusted by Bonferroni correction to address Type I error due to multiple comparisons

*CS7* Compressed SENSE acceleration factor 7, *CS10* CS acceleration factor 10, *CS10-AI* CS10 with adaptive-CS-net reconstruction

**Fig. 2:**
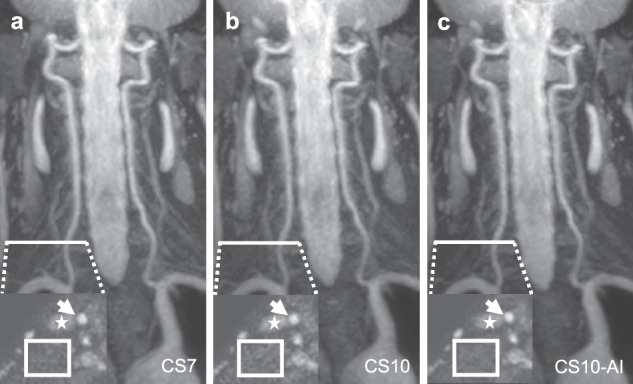
Vertebral arteries of a 59-year-old male volunteer (maximum intensity projection in coronal plane, 25 mm slice thickness). Insert shows axial source images (voxel size = 0.67 × 0.67 × 0.75 mm^3^) at the V1 segment of the right vertebral artery as indicated by the white line. Background noise (square) is more present in compressed SENSE acceleration factor 10 (CS10; **b**) and less present in CS10 with adaptive-CS-Net reconstruction (CS10-AI; **c**) compared to CS7 (**a**). Vessel contours of the common carotid arty (arrow) and external jugular vein (asterisk) are less clearly delineated in CS10 and more clearly delineated in CS10-AI compared to CS7

**Fig. 3:**
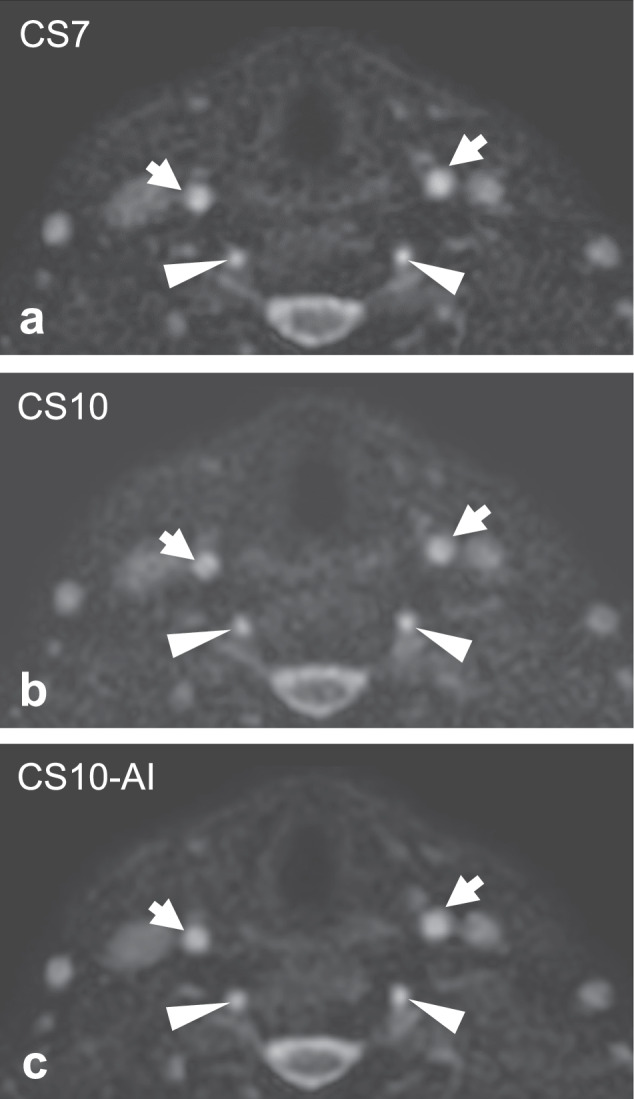
Axial source images (voxel size = 0.67 × 0.67 × 0.75 mm^3^) of a 66-year-old female volunteer. While higher image noise leads to decreased signal intensity and vessel wall delineation of the common carotid arteries (arrows) and the V2 segment of the vertebral arteries (arrowheads) in compressed SENSE acceleration factor 10 (CS10; **b**), CS10 with Adaptive-CS-Net reconstruction (CS10-AI; **c**) provides superior image quality which is comparable to CS7 (**a**)

Representative images are shown in Figs. 2 and 3.

#### Anatomical variants

Hypoplastic vertebral arteries (8.82% [3 of 34]) and a common origin of the carotid arteries (14.71% [5 of 34]) were found equally in CS7, CS10, and CS10-AI by both readers. No further vascular findings were reported.

#### Fat–water swapping artifacts

Seven volunteers (20.59%) showed a fat–water swapping artifact, all affecting the left subclavian artery. In one case, the artifact was only present in CS7, while CS10 and CS10-AI showed no artifact. In all other cases, the artifacts were present to the same extent in CS7, CS10, and CS10-AI. All artifacts could be clearly differentiated from true morphological features by a characteristic focal signal decrease in the water-only map and a corresponding focal signal increase in the fat-only map.

### Objective image quality

**Fig. 4:**
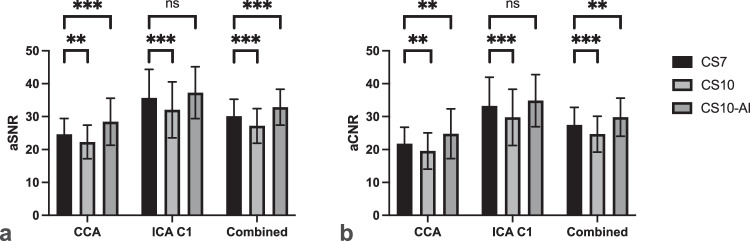
**a** aSNR and (**b**) apparent aCNR calculated for the common carotid arteries (CCA) and the C1 segment of the ICA C1, as well as both segments combined (all values are averaged across the right and left carotid arteries). Compared to compressed SENSE acceleration factor 7 (CS7), CS acceleration factor 10 (CS10) achieved lower aSNR and aCNR values, while CS10 with adaptive-CS-Net reconstruction (CS10-AI) yielded higher results. Only the C1 segment of the ICA showed no significant difference between CS7 and CS10-AI. *p*-values (paired *t*-test) were adjusted by Bonferroni correction to address Type I error due to multiple comparisons. ns: *p* > 0.05; ^**^p < 0.01; ^***^p < 0.001

Overall, CS10 achieved lower aSNR and aCNR than CS7 (aSNR: 27.17 ± 5.28 *versus* 30.12 ± 5.14; *p* < 0.001 and aCNR: 24.67 ± 5.44 *versus* 27.49 ± 5.34; *p* < 0.001) while CS10-AI yielded higher aSNR and aCNR than CS7 (aSNR: 32.86 ± 5.47 *versus* 30.12 ± 5.14; *p* < 0.001 and aCNR: 29.81 ± 5.79 *versus* 27.49 ± 5.34; *p* = 0.001). Further details are provided in Table 5 and corresponding plots are shown in Fig. 4.

**Table 5:** Comparison of objective image quality

	CS7	CS10	*p*	CS10-AI	*p*
aSNR (mean *±* SD)					
CCA	24.6 ± 4.85	22.29 ± 5.11	**0.003**	28.44 ± 7.12	**<** **0.001**
ICA C1	35.64 ± 8.73	32.05 ± 8.52	**<** **0.001**	37.27 ± 7.89	0.115
Combined	30.12 ± 5.14	27.17 ± 5.28	**<** **0.001**	32.86 ± 5.47	**<** **0.001**
aCNR (mean *±* SD)					
CCA	21.75 ± 5.02	19.56 ± 5.49	**0.003**	24.78 ± 7.57	**0.001**
ICA C1	33.24 ± 8.74	29.77 ± 8.53	**<** **0.001**	34.84 ± 7.92	0.120
Combined	27.49 ± 5.34	24.67 ± 5.44	**<** **0.001**	29.81 ± 5.79	**0.001**

Bold indicates statistical significance. *p*-values (paired *t*-test) were adjusted by Bonferroni correction to address Type I error due to multiple comparisons

*CCA* Common carotid artery, *CS7* Compressed SENSE acceleration factor 7, *CS10* CS acceleration factor 10, *CS10-AI* CS10 with adaptive-CS-net reconstruction, *ICA* C1 extracranial segment of the internal carotid artery, *SD* Standard deviation

The ERD was longer in CS10 compared to CS7 (2.28 ± 0.71 mm *versus* 1.96 ± 0.58 mm; *p* = 0.004) while there was no significant difference between CS10-AI and CS7 (2.07 ± 0.69 mm *versus* 1.96 ± 0.58 mm; *p* = 0.776).

## Discussion

In this prospective, single-center study, for the first time, the feasibility of sub-1-min 3D isotropic flow-independent non-contrast MRA (REACT) of the extracranial arteries was demonstrated using a novel DL-based reconstruction algorithm (CS-AI). This innovation represents a relevant advancement in the field of vascular imaging, particularly for time-sensitive cases such as stroke evaluations or other acute conditions, allowing for quicker clinical decision-making and potentially expedited treatment. Furthermore, these shortened scan times could improve patient throughput.

The major findings of this study are the following:

1. CS-AI reconstruction enables the acquisition of non-contrast MRA in a short scan time of only 0:55 min:s without compromising image quality, representing a 31.3% reduction compared to the current clinical standard CS7;
2. acceleration of REACT without DL-based reconstruction causes significantly lower subjective and objective image quality compared to the clinical standard and CS-AI; and
3. CS10-AI accelerated REACT even exceeds in part the clinical standard using CS7 regarding subjective and objective image quality.

The subjective image quality of REACT aligns with the findings of previous studies investigating REACT using solely CS for acceleration of image acquisition. In the current study, a median of 5.07 [4.52–5.43] out of 7 was found for CS7, 4.71 [4.38–5.11] out of 7 for CS10 and 5.29 [4.88–5.43] out of 7 for CS10–AI, which is comparable to Gietzen et al reporting 4 [3, 4] out of 5 for CS7 [17] and Hoyer et al achieving 5 [4, 5] out of 5 for CS4 [16]. The grading of the different vessel segments was also analogous, with the ICA obtaining the highest scores, while the vertebral arteries were rated as mediocre [15, 17]. In the present study, CS10-AI showed to CS7 superior overall image quality, primarily due to its results in the aortic arch. This might be attributed to the shorter scan time, which reduces the susceptibility to pulsation artifacts. In previous studies conducted by Pennig et al [15] (CS4) and Gietzen et al (CS7) [17], no difference was observed regarding the aortic arch between CE-MRA and REACT. Thus, the enhanced image quality achieved by CS-AI further underlines the potential of REACT as a valid non-contrast alternative. The image quality scores were in line with the pairwise forced choice comparison, which revealed no preference between the CS7 and CS10-AI, while both were preferred over CS10.

Fat–water swapping artifacts can occur in Dixon-based reconstructions due to B_0_ inhomogeneities, especially at higher field strengths [28]. In the present study, 20.6% of patients showed these artifacts affecting only the left subclavian artery without any difference between CS and CS-AI. These results are lower than in previous studies using CS4 (28.6–34.3%) [15, 16] and CS 7 (35.5%) [17], in which these artifacts also affected the left CCA. The lower occurrence in the present study despite using even higher CS factors is mostly due to different MRI systems employed, as the current study was performed on a novel system with improved gradient performance. However, by acquiring the fat-suppressed images alongside the fat-only maps, these artifacts can be easily identified and distinguished from true morphological features.

Regarding image noise, CS10-AI was subjectively perceived as less impaired and CS10 as more impaired than CS7, which aligns with the objective results for aSNR and aCNR. However, the aSNR and aCNR of CS7 were below the values reported by Gietzen et al [17] using the same acceleration factor (*e.g.*, overall aCNR 27.5 ± 5.3 *versus* 49.4 ± 15.0), which could be attributed to minor discrepancies in methodology or the use of a different MRI scanner. To further enhance the objective evaluation of image quality, the ERD demonstrated no loss of edge sharpness of the right CCA when CS-AI was employed, which is consistent with the findings of Bischoff et al [26] for the prostate fascia in an accelerated T2-weighted sequence and the findings of Koktzoglou et al [29] for the CCA in an accelerated “quiescent interval slice-selective” (QISS)-MRA using a different DL-algorithm.

This study aligns with previous research on acceleration using CS-AI for cardiovascular imaging. Pednekar et al [20] accelerated the acquisition of cardiac cine balanced steady-state free precession (bSSFP) at 1.5 T from SENSE 2 to CS5-AI and reduced the breath-hold time by 57% while maintaining diagnostic image quality. Wu et al [21] used CS6-AI for the acceleration of non-contrast 3-T MRA of the coronary arteries and reduced the acquisition time by more than 50% compared to the preliminary work of Haman et al using SENSE factor 1.5 [30] while yielding high diagnostic accuracy for coronary artery stenosis compared to CTA. CS-AI has also shown advancements in musculoskeletal radiology. Dratsch et al [31] demonstrated a 54% reduction in scan time for a fat-saturated 3D proton density-weighted sequence using CS10-AI of the knee compared to SENSE factor 2, while Fervers et al [32] have reported a 65% reduction in scan time for T2-weighted 3D MRI of the spine employing CS8-AI compared to SENSE 2.5.

Other non-contrast MRA techniques for the imaging of the extracranial arteries have already shown the potential of DL-based reconstruction. Scan times of 1:40 min:s for 3D thin-slab QISS-MRA [33] and 2:21 min for 2D QISS-MRA [29] were reported. Table 1 provides an overview of the scan parameters for selected non-contrast MRA sequences and conventional CE-MRA. Nevertheless, besides reconstruction times of up to 15:51 min:s, the required additional central processing unit cluster limits its feasibility for other centers [33]. In contrast, the CS-AI technique was fully integrated into the clinical system and does not require extensive reconstruction times (20 s) or postprocessing, while being performed with the standard hardware. Even though the CS-AI reconstruction time in this study is about 5-s longer than the standard CS reconstruction (15 s), this might not impact the clinical workflow. Future developments in hardware acceleration (*e.g*., graphics processing units−GPUs) or optimized algorithms could potentially further reduce reconstruction times.

Using CS10-AI, we enabled the acquisition of a non-contrast MRA of the neck being comparable if not faster than currently used CE-MRA sequences, which routinely last about 1 min and in fact even longer when considering patient preparation and bolus tracking [17]. This development offers several advantages, including a reduction in the likelihood of artifacts due to patient movement, as patients are required to remain still for a shorter period of time [34] and minimal loss of time if the sequence needs to be repeated. Consequently, time can be saved in emergency situations while patient comfort is enhanced [35]. The results may indicate the potential of CS-AI reconstruction for other nonvascular MRI protocols.

In future studies, the inclusion of patients with vascular pathologies, such as stenosis, could further assess how CS-AI reconstruction impacts artifact rates, diagnostic accuracy, and image quality. The incorporation of multiple neural networks in the image reconstruction chain (combining DL-based k-space to image space reconstruction as employed in the current work with so-called super-resolution DL networks, further increasing image sharpness) may reduce acquisition time and improve image quality even more [36]. These combinations have already been employed for two-dimensional imaging. Terzis et al achieved a 57% reduction in scan time for an entire knee protocol while maintaining image quality [37], while Bischoff et al demonstrated a reduction in scan time of up to 36% with enhanced image quality in T2-weighted prostate MRI.

### Limitations

First, a selection bias must be acknowledged since predominantly healthy volunteers do not align with the demographic profile of patients undergoing neck MRA in clinical practice. Consequently, the study lacked the inclusion of pathologies (*e.g*., stenosis or dissection). Additional studies are required to confirm the findings of this study. Second, no comparison of REACT was performed to digital subtraction angiography, which remains the reference standard in this setting. However, the primary purpose of (non-contrast) MRA in this context is not to replace digital subtraction angiography, but rather to serve as a fast, noninvasive tool to rule out major stenosis or occlusion. Similarly, no direct comparison to CE-MRA was conducted in this study. Nevertheless, prior studies indicated the high image quality and diagnostic performance of REACT compared to CE-MRA [15–17]. Third, SNR and CNR may be regarded as a limitation since acceleration techniques may influence the true SNR and CNR [38, 39]. However, to verify the subjective assessments of vessel quality and noise, we performed measurements of aSNR and aCNR which were largely consistent with subjective results, and introduced the ERD as an objective image sharpness parameter. Fourth, a reconstruction with CS10-AI from the raw data set as CS10 was not technically feasible due to software restrictions. Therefore, the threefold acquisition was conducted in succession, consequently hampering a direct comparison between CS10 and CS10-AI while posing the risk of motion artifacts given the required additional scan time. Consequently, a direct comparison with CS7 was the focus of this study. Five, the REACT acquisition was not randomized, potentially being disadvantageous for CS10 and CS10-AI sequences. Six, the European Conformity−CE-certification and U.S. FDA approval of the DL-based reconstruction software (SmartSpeed, Philips Healthcare) represent a major strength for clinical implementation. However, its proprietary nature limits availability to the vendor’s MRI systems, while the computational power required may pose challenges for institutions with limited access to advanced hardware or processing infrastructure.

In conclusion, the acceleration of REACT using CS-AI enables for the first time the acquisition of cervical non-CE-MRA in less than one minute without a loss in subjective or objective image quality compared to the standard CS accelerated acquisition. These results yield practical benefits, including expedited clinical decision-making in time-sensitive cases like ischemic stroke, reduced scan time costs, and enhanced patient comfort, making it highly relevant for clinical use. Further studies on a patient population with pathologies are required to confirm these findings.

## Data Availability

The datasets used and/or analyzed during the current study are available from the corresponding author upon reasonable request.

## Abbreviations

3D: Three-dimensional
aCNR: Apparent contrast-to-noise ratio
aSNR: Apparent signal-to-noise ratio
CCA: Common carotid artery
CE-MRA: Contrast-enhanced magnetic resonance angiography
CS: Compressed SENSE
CS10: REACT with acceleration factor 10 acquired without DL-based reconstruction
CS10-AI: REACT with acceleration factor 10 with DL-based reconstruction
CS7: REACT with acceleration factor 7 acquired without DL-based reconstruction
CS-AI: Compressed SENSE with adaptive intelligence reconstruction
DL: Deep learning
ERD: Edge-rise distance
ICA: Internal carotid artery
MRA: Magnetic resonance angiography
MRI: Magnetic resonance imaging
QISS: Quiescent interval slice-selective
REACT: Relaxation-enhanced angiography without contrast and triggering
SENSE: Sensitivity encoding
